# The Potential of Wearable Limb Ballistocardiogram in Blood Pressure Monitoring via Pulse Transit Time

**DOI:** 10.1038/s41598-019-46936-9

**Published:** 2019-07-23

**Authors:** Peyman Yousefian, Sungtae Shin, Azin Mousavi, Chang-Sei Kim, Ramakrishna Mukkamala, Dae-Geun Jang, Byung-Hoon Ko, Jongwook Lee, Ui Kun Kwon, Youn Ho Kim, Jin-Oh Hahn

**Affiliations:** 1 Department of Mechanical Engineering, University of Maryland, College Park, MD USA; 2 School of Mechanical Engineering, Chonnam National University, Gwangju, Korea; 3 Department of Electrical and Computer Engineering, Michigan State University, East Lansing, MI USA; 4 Device & System Research Center, Samsung Advanced Institute of Technology, Suwon, Gyeonggi Korea

**Keywords:** Cardiovascular biology, Cardiovascular biology

## Abstract

The goal of this study was to investigate the potential of wearable limb ballistocardiography (BCG) to enable cuff-less blood pressure (BP) monitoring, by investigating the association between wearable limb BCG-based pulse transit time (PTT) and BP. A wearable BCG-based PTT was calculated using the BCG and photoplethysmogram (PPG) signals acquired by a wristband as proximal and distal timing reference (called the wrist PTT). Its efficacy as surrogate of BP was examined in comparison with PTT calculated using the whole-body BCG acquired by a customized weighing scale (scale PTT) as well as pulse arrival time (PAT) using the experimental data collected from 22 young healthy participants under multiple BP-perturbing interventions. The wrist PTT exhibited close association with both diastolic (group average r = 0.79; mean absolute error (MAE) = 5.1 mmHg) and systolic (group average r = 0.81; MAE = 7.6 mmHg) BP. The efficacy of the wrist PTT was superior to scale PTT and PAT for both diastolic and systolic BP. The association was consistent and robust against diverse BP-perturbing interventions. The wrist PTT showed superior association with BP when calculated with green PPG rather than infrared PPG. In sum, wearable limb BCG has the potential to realize convenient cuff-less BP monitoring via PTT.

## Introduction

Hypertension is known to raise the risk of cardiovascular disease and stroke. Recent statistics indicate that hypertension is prevalent: approximately 1/3 of adults in the United States have high blood pressure (BP); approximately 20% of hypertensives are not even aware of high BP; and only approximately half of those aware of high BP have their BP under control^1^. Hypertension is a silent killer – it does not accompany any warning sign and/or symptoms for death. Hence, frequent measurement of BP is highly desirable for early detection and treatment of hypertension. However, state-of-the-art BP measurement techniques widely used in healthcare – auscultation^2^ and oscillometry^3^ – are not amenable for convenient frequent BP monitoring: the former requires a trained operator, while both require cumbersome occlusive cuff. Hence, there has been an increasing interest in developing innovative techniques and systems for convenient BP monitoring that are operator-less and cuff-less.

One of the most widely pursued operator-less and cuff-less BP monitoring techniques is based on the pulse transit time (PTT) principle^4^. PTT is the time required for an arterial wave (e.g., BP) to travel from one (usually proximal) arterial site to another (usually distal) and is known to be inversely associated with BP via nonlinear pressure-area relationship of the arterial wall^4^. Due to the inconvenience associated with the acquisition of proximal arterial pulse signals, the vast majority of existing PTT-based BP monitoring studies have resorted to pulse arrival time (PAT) in which the R wave of the electrocardiogram (ECG) is used as the proximal timing reference^4^. PAT has shown its efficacy for association with systolic BP (SP) in many previous investigations^4^. However, PAT is composed of PTT and pre-ejection period (PEP), which does not vary consistently in response to BP. In fact, our prior work has suggested that the efficacy of PAT may be degraded under BP-perturbing interventions in which PTT and PEP vary in the opposite directions, due to the mutual cancellation of their respective changes in response to BP^5,6^. To overcome the drawback of PAT, the ability for convenient acquisition of arterial pulse signals as proximal timing reference for PTT is desired.

Ballistocardiogram (BCG) is the measurement of body movement in response to heartbeat^7^. With the recent advances in electronics and sensing technologies, the acquisition of the BCG is becoming more and more convenient; a number of BCG-measuring instruments assuming a wide range of form factors have been proposed in the literature, including bed^8,9^, chair^10,11^, scale^12–14^, and wearables^15^. In addition, the BCG is known to be closely associated with arterial BP^16^. Hence, the BCG has the potential to offer convenient options for the acquisition of proximal arterial pulse signals for calculating PTT. In fact, our prior work suggests that characteristic features extracted from the BCG acquired with a scale-like platform (i.e., a high-performance force plate) have the potential for convenient cuff-less BP monitoring^5,6^.

Scale-like platforms measure the resultant force acting on the main torso or the resulting movement of the whole body (called the whole-body BCG), depending on the sensors embedded in the instrument^17^. Given that it is the direct response to the force exerted by the blood ejected by the heart, the whole-body BCG provides rich insights on the arterial BP^16^. The whole-body BCG is also transferred to the limb sites (e.g., wrist and upper arm) to elicit the movement of the limbs (called the limb BCG)^18^. Compared with the whole-body BCG, the limb BCG is much more amenable to ultra-convenient measurement with wearable devices such as wristband. However, its morphology is largely distinct from the whole-body BCG due to the compliant nature of the musculoskeletal system as well as the discrepancy in the instrument type^6,19^. In fact, even the typical shape of the limb BCG and the available features therein remain mysterious with very limited prior work. Hence, cuff-less BP monitoring based on wearable limb BCG presents a formidable challenge despite the remarkable success associated with the whole-body BCG. Regardless, to the best of our knowledge, no prior work has rigorously examined the opportunities for ultra-convenient cuff-less BP monitoring with wearable limb BCG.

In our prior work, we elucidated the physical implications of the whole-body BCG due to the force exerted by the blood ejected by the heart: that it originates from the BP gradients in the ascending and descending aorta^16^. Based on the physical insights thus garnered, we demonstrated that characteristic features in the whole-body BCG acquired using a high-performance force plate can serve as viable surrogates of BP^5,6^. We also illustrated that the shape of the whole-body BCG may exhibit variability depending on the type of the measurement instrument^17^, which may be generalizable to the limb BCG. Hence, our prior work provides strong motivation to extend the potential of the whole-body BCG to the more convenient limb BCG for cuff-less BP monitoring.

Inspired by our prior success with the whole-body BCG and the ultra-convenience of wearable limb BCG, the goal of this study was to investigate the potential of wearable limb BCG to enable cuff-less BP monitoring via PTT, by investigating the association between wearable limb BCG-based PTT and BP in comparison with whole-body BCG-based PTT and PAT. To this aim, a wearable BCG-based PTT was calculated using the BCG and photoplethysmogram (PPG) signals acquired by a wristband as proximal and distal timing reference (called the wrist PTT). Its efficacy as surrogate of BP was examined in comparison with PTT calculated using the whole-body BCG acquired by a customized weighing scale (scale PTT) as well as PAT using the experimental data collected from 22 young healthy participants under multiple BP-perturbing interventions. The primary novelty of the present work distinct to our prior work is that it shows the potential of limb BCG for wearable BP monitoring. Specifically, it elucidated the typical shape of the wrist BCG waveform, and it also demonstrated the wrist BCG features relevant to PTT calculation with physical justification. The secondary novelty of the present work is that it illustrated the feasibility of using commercial low-cost weighing scale for acquisition of the BCG for PTT-based BP monitoring.

## Methods

In this study, we investigated the association between PTT based on a wearable limb BCG (wrist BCG) and BP by the following steps: (i) requisite signals to calculate PTT based on both whole-body BCG and wrist BCG as well as PAT, and the corresponding reference systolic (SP) and diastolic (DP) BP were acquired from the study participants under an array of BP-perturbing interventions; (ii) requisite features to calculate PTT and PAT were extracted from the acquired signals, and then PTT and PAT were calculated; and (iii) the association between the PTT and PAT thus calculated versus reference BP was analyzed. Details follow.

### Data acquisition

Under the approval of the Institutional Review Board (IRB) of the University of Maryland and written informed consent, 22 young healthy volunteers were studied while strictly abiding by the guidance of the IRB. The study details may be found in our prior work^20^. Tables 1 and 2 summarize the demographics and ethnicity of the study participants, respectively.

**Table 1:** Demographics of the study participants (mean +/− SD).

Age [Yr]	Gender	Weight [kg]	Height [cm]
24 +/− 5	M: 16, F: 6	73 +/− 16	174 +/− 8

**Table 2:** Ethnicity of the study participants.

Hispanic Latin	2
American Indian/Alaska Native	0
Black/African American	1
Asian	5
Native Hawaiian/Pacific Islander	0
White	11
Unknown	3

**Figure 1:**
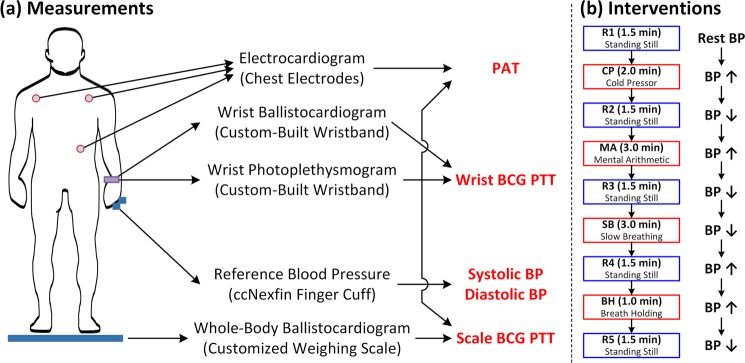
Data acquisition set-up and BP-perturbing interventions. PAT: pulse arrival time. PTT: pulse transit time. BCG: ballistocardiogram. BP: blood pressure. R1, R2, R3, R4, R5: rest states. CP: cold pressor. MA: mental arithmetic. SB: slow breathing. BH: breath holding.

From each participant, the following waveform signals were acquired: (i) an ECG measured with 3-gel electrodes in the Lead II configuration and interfaced with a wireless amplifier (BN-EL50, Biopac Systems, Goleta, CA, USA); (ii) a whole-body BCG measured as body displacement using a strain gauge embedded in a customized commercial weighing scale (BC534, Tanita, Tokyo, Japan); (iii) a wearable limb BCG measured using a MEMS accelerometer embedded in an in-house manufactured wristband; (iv) green and infrared (IR) PPG measured using in-house manufactured reflectance mode PPG sensors also embedded in the wristband; and (v) a reference BP pulse measured with a fast servo-controlled finger cuff embedded with a blood volume waveform sensor on the ring finger of a hand to implement the volume clamp method, which was height-compensated and transformed to brachial BP pulse (ccNexfin, Edwards Lifesciences, Irvine, CA, USA) (Fig. 1(a)). All the devices other than the wristband were interfaced to a laptop computer through a data acquisition unit (MP150, Biopac Systems, Goleta, CA, USA) to record the ECG, whole-body BCG, and reference BP pulse waveforms at 1 kHz sampling rate. The wristband was interfaced directly to the laptop computer via a custom-built software to record the wearable limb BCG and the PPG waveforms at 250 Hz sampling rate. Data acquisition was synchronized via an analog square pulse signal sent by MP150 to the wristband so that all the recordings started simultaneously and the wristband recordings were performed exactly at the moment when 4 MP150 recordings were performed.

The above waveform signals were acquired during four BP-perturbing interventions while the participants stood on the scale (Fig. 1(b)). Each participant stood still for 1.5 min for an initial rest state (R1). Then, the participant performed the cold pressor (CP) intervention for up to 2 min, in which the participant was asked to immerse his/her free hand into ice water. Followed by standing still for 1.5 min for a second rest state (R2), the participant conducted the mental arithmetic (MA) intervention for up to 3 min, in which the participant was asked to repeatedly add the digits of a three-digit number and add the sum to the original number. Followed by standing still for 1.5 min for a third rest state (R3), the participant conducted the slow breathing (SB) intervention for up to 3 min, in which the participant was asked to repeatedly take slow and deep breaths. Followed by standing still for 1.5 min for a fourth rest state (R4), the participant conducted the breath holding (BH) intervention, in which the participant was asked to hold his/her breath after normal exhalation (i.e., starting from functional residual capacity). Finally, the participant stood still for 1.5 min for a fifth rest state (R5). Throughout the course of the study, the participants were asked to minimize movement with their arms placed at the side in the standing posture. The acquisition was made continuously throughout the study.

### Calculation of PTT and PAT

**Figure 2:**
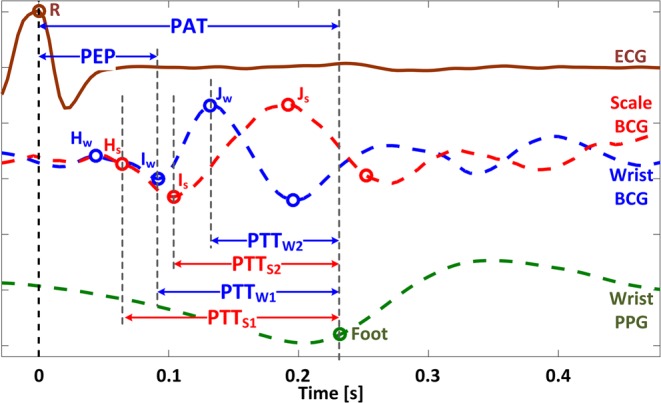
Features extracted from ECG (R wave), BCG (H_S_, I_S_, J_S_, H_W_, I_W_, J_W_ waves), and PPG (foot) signals, as well as scale PTT (PTT_S1_, PTT_S2_), wrist PTT (PTT_W1_, PTT_W2_), and PAT. Only green PPG signal is shown for the sake of illustration. PAT: pulse arrival time. PEP: pre-ejection period. PTT: pulse transit time. ECG: electrocardiogram. BCG: ballistocardiogram. PPG: photoplethysmogram. H_S_, I_S_, J_S_: H, I, and J waves in the scale BCG. H_W_, I_W_, J_W_: H, I, and J waves in the wrist BCG.

From the measured signals, PTT based on the scale and the wrist BCG as well as PAT were calculated based on the characteristic features extracted from the BCG, PPG, and ECG signals (Figs 1(a) and 2). Details follow.

The collected data were first down-sampled to 250 Hz. For each participant, the data were segmented into nine periods: R1, CP, R2, MA, R3, SB, R4, BH, and R5. In each period, the signals were processed as follows. First, the ECG R waves were detected as the local peaks in the ECG signal. Second, the scale and wrist BCG as well as PPG signals were band-pass filtered using a 1^st^-order Butterworth filter with nominal pass band of 0.5 Hz~15 Hz (in other words, the pass band of individual participants were empirically varied around the nominal pass band to effectively remove participant-specific drifts and artifacts due to respiration and movement). Third, the BCG and PPG beats were identified with the ECG gating. Fourth, individual beats were visually inspected and those associated with corrupted BCG (e.g., large-amplitude BCG due to non-negligible motion artifacts) and PPG (e.g., small signal-to-noise ratio PPG due to low-quality sensor-skin contact) signals were excluded from further analysis. Fifth, the scale and wrist BCG signals were smoothed using a causal 8-beat exponential moving average filter to suppress residual motion artifacts. Sixth, the PPG foot was extracted using the intersecting tangent method^5,21^. Seventh, characteristic features (called the waves; Fig. 2) consistently available in the scale and wrist BCG signals were identified, which were then extracted from each beat as follows: (1) the scale J_S_ and wrist J_W_ waves were identified as the local maximum in the initial 15~40% window of the beat; (2) the scale I_S_ and wrist I_W_ waves were identified as the nearest local negative waves before the J_S_ and J_W_ waves, respectively; and (3) the scale H_S_ and wrist H_W_ waves were identified as the nearest local positive waves before the I_S_ and I_W_ waves, respectively. The local waves were identified as local extrema, except for the H_S_ and I_W_ waves which were identified based on the intersecting tangent method. The H_S_ wave was identified as the intersection between the horizontal line passing the nearest local positive peak before the I_S_ wave and the tangential line passing the point of the maximum negative slope in the interval between the I_S_ wave and the nearest local positive peak before the I_S_ wave. The I_W_ wave was identified as the intersection between the horizontal line passing the nearest local negative peak before the J_W_ wave and the tangential line passing the point of the maximum positive slope in the interval between the J_W_ wave and the nearest local negative peak before the J_W_ wave.

From the consistently available features thus extracted, PTT and PAT were calculated as follows (Fig. 2). First, two scale PTTs were calculated as whole-body BCG-based PTT using the scale BCG and the wrist PPG as the proximal and distal timing references: PTT_S1_ as the interval between the H_S_ wave and the PPG foot, and PTT_S2_ as the interval between the I_S_ wave and the PPG foot. Second, two wrist PTTs were likewise calculated as wrist BCG-based PTT using the wrist BCG and the wrist PPG as the proximal and distal timing references: PTT_W1_ as the interval between the I_W_ wave and the PPG foot, and PTT_W2_ as the interval between the J_W_ wave and the PPG foot. Third, PAT was calculated as the interval between the ECG R wave and the PPG foot. To examine the influence of the choice of the PPG signal on the efficacy of PTT and PAT, all the above PTTs and PAT were calculated using green and IR PPG signals, resulting in eight PTTs and two PATs for analysis. In addition to the above PTT and PAT, pre-ejection period (PEP) was calculated as the interval between the ECG R wave and the I_W_ wave similarly to our prior work^5^.

### Analysis of association between PTT and PAT versus BP

To analyze the association between PTT and PAT versus BP, the reference DP and SP were extracted from each beat as the minimum (DP) and maximum (SP) of the height-compensated brachial BP signal. In addition, in each rest or intervention period (i.e., R1, CP, R2, MA, R3, SB, R4, BH), beat-to-beat fluctuations in the scale PTT, wrist PTT, and PAT were suppressed as follows: (i) outliers in the beat-to-beat sequences of the scale PTT, wrist PTT, and PAT (defined as the PTT and PAT values outside of +/−3 × SD from its mean value within each period) were removed; and (ii) the beat-to-beat sequences were subsequently smoothed using a quadratic Savitzky-Golay filter (which is well suited to filtering of non-uniformly spaced sequences)^22^. Then, the efficacy of the scale and wrist PTT as well as PAT in association with BP was analyzed using the entire data as well as using subsets of the data associated with individual rest-intervention period pairs (i.e., R1-CP, R2-MA, R3-SB, and R4-BH). Details follow.

First, the association between PTT and PAT versus BP was analyzed using the entire data, specifically in the maximum BP change regimes associated with all the rest and intervention periods. The goal of the analysis was to comparatively assess the overall efficacy of scale PTT, wrist PTT, and PAT as surrogate of BP across a wide range of BP changes and BP-perturbing interventions. In each period, the instant at which the reference BP attained extremum (minimum for rest and SB periods, and maximum for CP, MA, and BH periods) was identified. Then, the reference BP as well as the scale PTT, wrist PTT, and PAT representative of the period were determined as their respective median values within the five-beat interval around the extremum. Subsequently, up to nine pairs of reference BP and the corresponding scale PTT, wrist PTT, and PAT associated with 9 periods (R1, CP, R2, MA, R3, SB, R4, BH, and R5) were obtained from each participant. Considering that the pair of reference BP and the corresponding scale PTT, wrist PTT, and PAT could not be obtained in a subset of periods (especially BH due to the limited number of beats available from the collected data), only the participants equipped with the pair of reference BP and the corresponding PTT and PAT for ≥3 rest-intervention period pairs (R1-CP, R2-MA, R3-SB, and R4-BH; thus ≥6 periods in total) were included for analysis of the overall PTT-BP and PAT-BP associations.

Second, the association between the wrist PTT and PAT versus BP was analyzed using subsets of the data, specifically in the individual rest-intervention period pairs (R1-CP, R2-MA, R3-SB, and R4-BH). The goal of the analysis was to examine the robustness and consistency of the association between the wrist PTT and PAT versus BP across diverse BP-perturbing interventions. In each rest-perturbation period pair, the range of BP was segmented into 1 mmHg bins, and the median wrist PTT and PAT values contained in each bin were calculated. Then, the reference BP and the corresponding wrist PTT and PAT thus segmented were employed for analysis of the intervention-specific wrist PTT-BP and PAT-BP associations.

The efficacy of the scale PTT, wrist PTT, and PAT in their association with BP was quantified in terms of three metrics: correlation coefficient, root-mean-squared error (RMSE), and mean absolute error (MAE) between reference BP and BP calibrated from the scale PTT, wrist PTT, and PAT. These metrics were first computed in each participant and then summarized as mean and standard error (SE) across all participants. The Bland-Altman analysis was also conducted to assess the limits of agreement between reference and calibrated BP. In the analysis, the calibrated BP was computed in each participant as follows. In case of the analysis for the entire data in the maximum BP change regimes, linear regression models that relate each PTT (PTT_S1_, PTT_S2_, PTT_W1_, and PTT_W2_, all in conjunction with both green and IR PPG signals) and PAT (in conjunction with both green and IR PPG signals) to reference DP and SP were calculated using the data associated with ≥6 available rest and intervention periods, and the calibrated DP and SP were computed by inputting each PTT and PAT to the respective linear regression models. Subsequently, the correlation coefficients, RMSEs, and MAEs between reference versus calibrated DP and SP were computed as measures of the best-case association (where the best-case association means the PTT-BP and PAT-BP associations after participant-specific calibration using the linear regression model derived from all the available data). The significance in difference between wrist PTT and PAT was determined using the paired t-test with the Bonferroni correction for multiple comparisons (i.e., 2 comparisons: PTT_W1_ versus PAT and PTT_W2_ versus PAT). As part of the analysis, the efficacy of PTT and PAT calculated with green and IR PPG signals was investigated in terms of the same quantitative metrics. In case of the analysis for the individual rest-intervention period pairs, linear regression models that relate the best-performing wrist PTT in the analysis using the entire data (i.e., in the maximum BP change regimes) and PAT to reference DP and SP were calculated using all the available data associated with the rest-intervention period pair, and the calibrated DP and SP were computed by inputting the best-performing PTT and PAT to the corresponding linear regression models. Then, the correlation coefficients, RMSEs, and MAEs between reference versus calibrated DP and SP were likewise computed as measures of the best-case association.

## Results

**Figure 3:**
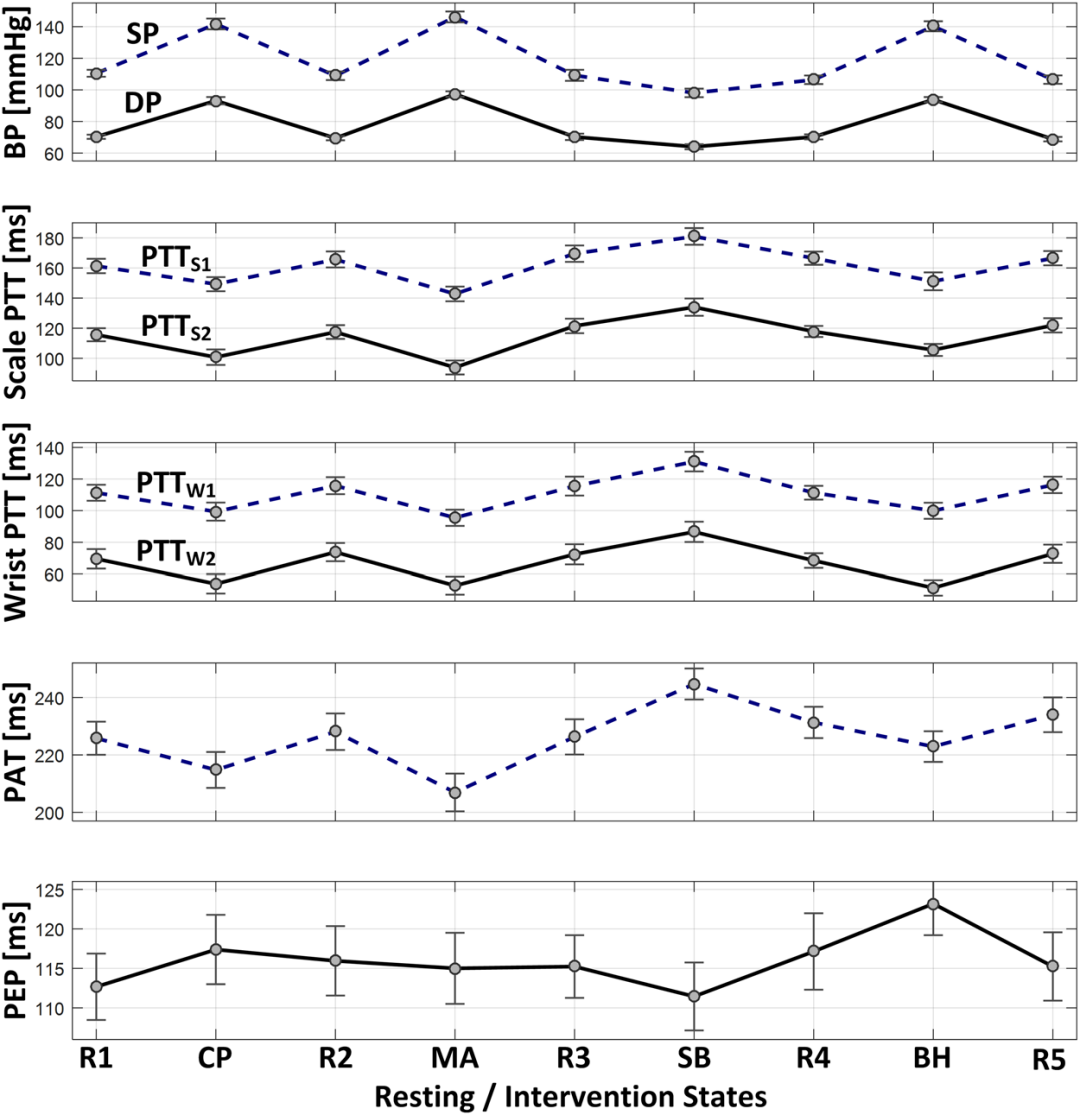
Changes in blood pressure (BP), scale pulse transit time (PTT) based on green photoplethysmogram (PPG), wrist PTT based on green PPG, pulse arrival time (PAT) based on green PPG, and pre-ejection period (PEP) in response to BP-perturbing interventions (mean +/− SE). SP: systolic BP. DP: diastolic BP. PTT_S1_: PTT calculated as time interval between H_S_ wave and PPG foot. PTT_S2_: PTT calculated as time interval between I_S_ wave and PPG foot. PTT_W1_: PTT calculated as time interval between I_W_ wave and PPG foot. PTT_W2_: PTT calculated as time interval between J_W_ wave and PPG foot.

**Figure 4:**
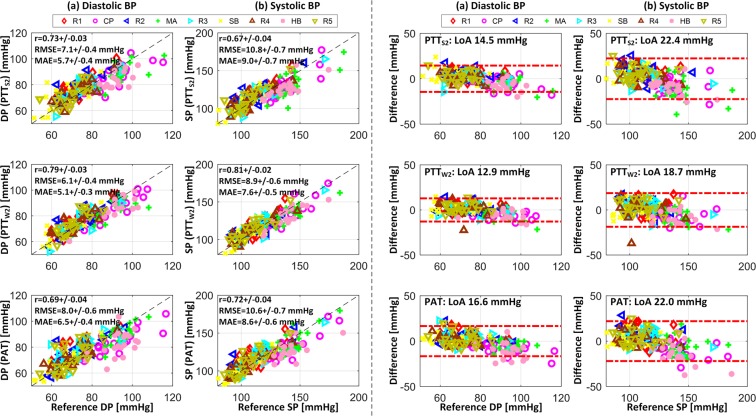
Correlation (left) and Bland-Altman (right) plots for reference blood pressure (BP) versus BP calibrated from scale pulse transit time (PTT) PTT_S2_, wrist PTT PTT_W2_, and pulse arrival time (PAT) based on green photoplethysmogram (PPG) in all participants. (**a**) Diastolic BP (DP). (**b**) Systolic BP (SP). r: correlation coefficient. RMSE: root-mean-squared error. MAE: mean absolute error. LoA: limits of agreement. R1, R2, R3, R4, R5: rest states. CP: cold pressor. MA: mental arithmetic. SB: slow breathing. BH: breath holding.

**Figure 5:**
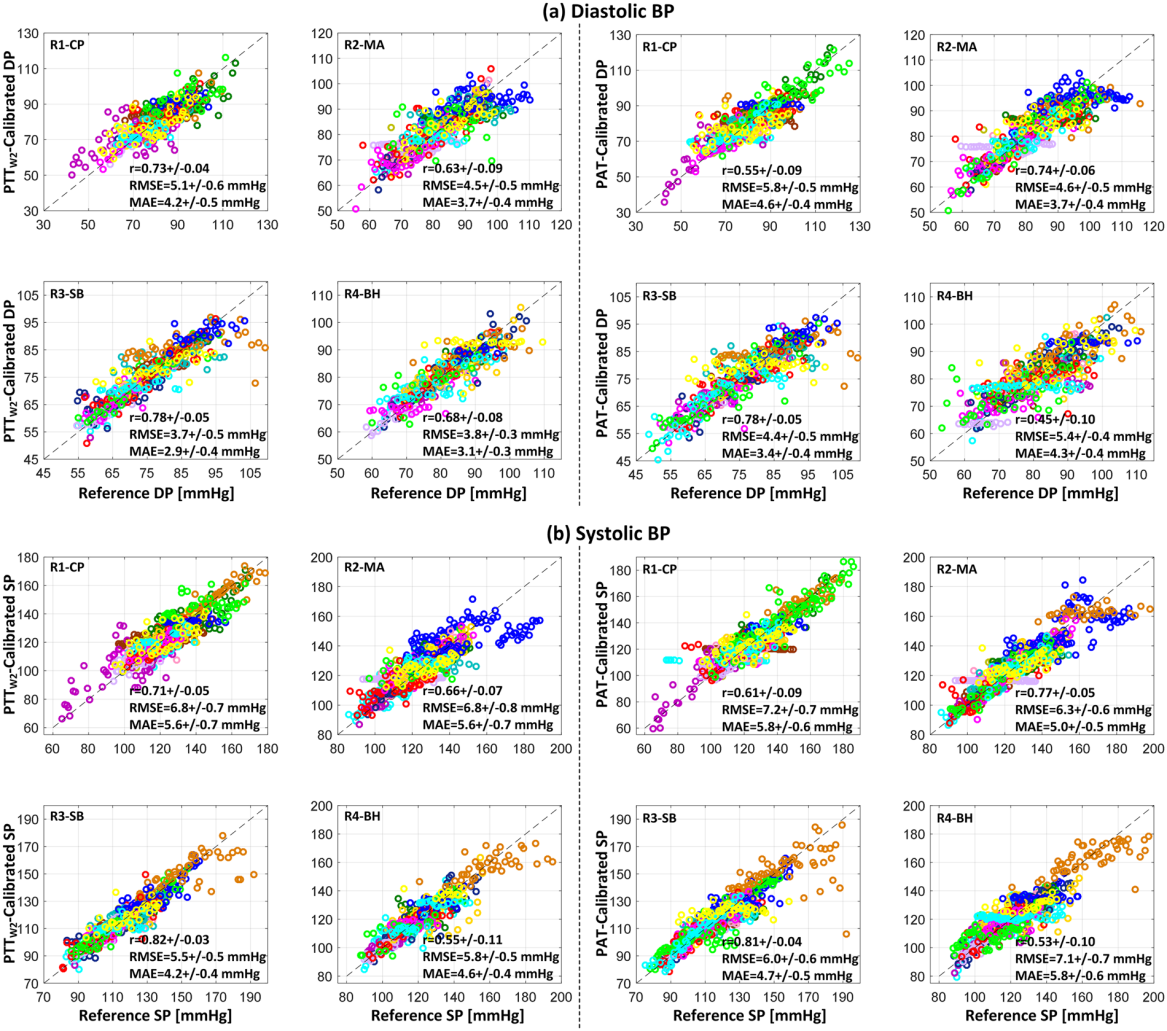
Reference blood pressure (BP) and BP calibrated from wrist pulse transit time (PTT) PTT_W2_ and pulse arrival time (PAT) based on green photoplethysmogram (PPG) in all participants associated with individual resting-intervention pairs. (**a**) Diastolic BP (DP). (**b**) Systolic BP (SP). r: correlation coefficient. RMSE: root-mean-squared error. MAE: mean absolute error. R1, R2, R3, R4, R5: rest states. CP: cold pressor. MA: mental arithmetic. SB: slow breathing. BH: breath holding.

Table 3 summarizes the resting BP levels and overall BP changes associated with the study participants. Table 4 shows the maximum changes in BP in response to each BP-perturbing intervention as well as all interventions. Figure 3 illustrates the changes in BP, scale and wrist PTT as well as PAT based on green PPG, and PEP in response to BP-perturbing interventions. Tables 5–7 summarize the correlation, RMSE, and MAE between reference BP versus BP calibrated from scale PTT, wrist PTT, and PAT based on green and IR PPG in all participants. Figure 4 illustrates the correlation, RMSE, and MAE as well as the Bland-Altman plots between reference BP versus BP calibrated from scale PTT PTT_S2_, wrist PTT PTT_W2_, and PAT based on green PPG in all participants. Figure 5 shows the correlation, RMSE, and MAE between reference BP versus BP calibrated from wrist PTT PTT_W2_ (which is the best-performing wrist PTT) and PAT (which is the most widely used conventional approach) based on green PPG in all participants associated with individual resting-intervention pairs.

**Table 3:** Resting BP levels and overall BP changes (mean +/− SE). DP: diastolic BP. SP: systolic BP.

	Resting Level [mmHg]	Overall Change [mmHg]
DP	78 +/− 1	38 +/− 2
SP	119 +/− 2	56 +/− 3

**Table 4:** BP changes in response to BP-perturbing interventions (mean +/− SE).

	R1→CP	CP→R2	R2→MA	MA→R3	R3→SB	SB→R4	R4→BH	BH→R5	Range
DP [mmHg]	+23 +/− 2	−24 +/− 2	+27 +/− 1	−26 +/− 2	−6 +/− 1	+6 +/− 1	+23 +/− 2	−25 +/− 2	38 +/− 2
SP [mmHg]	+31 +/− 3	−32 +/− 3	+37 +/− 2	−37 +/− 2	−10 +/− 2	+7 +/− 1	+33 +/− 3	−33 +/− 2	56 +/− 3

**Table 5:** Correlation between reference blood pressure (BP) versus BP calibrated from scale pulse transit time (PTT), wrist PTT, and pulse arrival time (PAT) (mean +/− SE). DP: diastolic BP. SP: systolic BP. ^ǂ^p < 0.025 with respect to PAT (paired t-test).

		DP		SP	
		Green PPG	IR PPG	Green PPG	IR PPG
Scale PTT	PTT_S1_	0.70 +/− 0.04	0.60 +/− 0.04	0.67 +/− 0.04	0.55 +/− 0.07
	PTT_S2_	0.73 +/− 0.03	0.59 +/− 0.05	0.67 +/− 0.04	0.54 +/− 0.07
Wrist PTT	PTT_W1_	0.75 +/− 0.03	0.61 +/− 0.07^ǂ^	0.76 +/− 0.03	0.61 +/− 0.08
	PTT_W2_	0.79 +/− 0.03^ǂ^	0.65 +/− 0.07^ǂ^	0.81 +/− 0.02^ǂ^	0.65 +/− 0.08
PAT		0.69 +/− 0.04	0.42 +/− 0.09	0.72 +/− 0.04	0.50 +/− 0.10

**Table 6:** Root-mean-squared error (RMSE) between reference blood pressure (BP) versus BP calibrated from scale pulse transit time (PTT), wrist PTT, and pulse arrival time (PAT) (mean +/− SE). DP: diastolic BP. SP: systolic BP. ^ǂ^p < 0.025 with respect to PAT (paired t-test).

[mmHg]		DP		SP	
		Green PPG	IR PPG	Green PPG	IR PPG
Scale PTT	PTT_S1_	7.2 +/− 0.5	7.5 +/− 0.4	10.9 +/− 0.8	10.8 +/− 0.5
	PTT_S2_	7.1 +/− 0.4	7.4 +/− 0.4	10.8 +/− 0.7	10.7 +/− 0.6
Wrist PTT	PTT_W1_	6.6 +/− 0.5^ǂ^	7.0 +/− 0.4^ǂ^	9.7 +/− 0.6	9.9 +/− 0.6
	PTT_W2_	6.1 +/− 0.4^ǂ^	6.5 +/− 0.5^ǂ^	8.9 +/− 0.6	9.6 +/− 0.8
PAT		8.0 +/− 0.6	9.0 +/− 0.6	10.6 +/− 0.7	11.2 +/− 1.0

**Table 7:** Mean absolute error (MAE) between reference blood pressure (BP) versus BP calibrated from scale pulse transit time (PTT), wrist PTT, and pulse arrival time (PAT) (mean +/− SE). DP: diastolic BP. SP: systolic BP. ^ǂ^p < 0.025 with respect to PAT (paired t-test).

[mmHg]		DP		SP	
		Green PPG	IR PPG	Green PPG	IR PPG
Scale PTT	PTT_S1_	5.8 +/− 0.3	6.2 +/− 0.3	9.1 +/− 0.7	9.0 +/− 0.5
	PTT_S2_	5.7 +/− 0.4	6.1 +/− 0.4	9.0 +/− 0.7	9.2 +/− 0.6
Wrist PTT	PTT_W1_	5.5 +/− 0.4	5.8 +/− 0.3^ǂ^	8.3 +/− 0.5	8.4 +/− 0.5
	PTT_W2_	5.1 +/− 0.3^ǂ^	5.3 +/− 0.4^ǂ^	7.6 +/− 0.5	8.0 +/− 0.7
PAT		6.5 +/− 0.4	7.4 +/− 0.5	8.6 +/− 0.6	9.4 +/− 0.9

## Discussion

Despite its potential for ultra-convenient BP monitoring with wide-ranging wearable devices, the limb BCG presents critical hurdles due to its unique yet unestablished morphology distinct from the relatively well understood whole-body BCG. To the best of our knowledge, no prior work has rigorously examined opportunities for the wearable limb BCG-based PTT and BP monitoring. In our initial attempt to tackle this challenge, this study intended to investigate the feasibility of ultra-convenient BP monitoring based on the PTT principle implemented using the BCG and PPG signals obtained from a single wrist-worn device. Our primary interests were (i) the overall association between the wrist PTT and BP with physiological justification and its consistency and robustness against diverse perturbations in BP; (ii) comparative performance between the wrist PTT and PAT in association with BP, and (iii) the effect of the choice of PPG on the association between wrist PTT and BP.

### BP changes in response to interventions

Both DP and SP of all the participants could be varied widely with the BP-perturbing interventions employed in this study. On the average, 38 mmHg change in DP and 56 mmHg change in SP were observed (Table 3), while all the participants underwent the minimum changes of >24 mmHg in DP and >37 mmHg in SP. Each intervention contributed comparably to these overall BP changes with >23 mmHg change in DP and >31 mmHg change in SP on the average, except SB which decreased both DP and SP only modestly (Table 4). Further, cardiovascular mechanisms responsible for the BP changes due to each of the BP-perturbing interventions was distinct: (1) CP increased BP via an increase in heart rate and total peripheral resistance; (2) MA increased BP via a large increase in heart rate (with the associated decrease in stroke volume) and total peripheral resistance; (3) SB decreased BP via a decrease in stroke volume; and (4) BH increased BP via a large increase in total peripheral resistance despite a notable decrease in heart rate, stroke volume, and cardiac output (not shown). To a large extent, the cardiovascular mechanisms observed in this study accord with the findings from prior work: CP^23–29^, MA^23,27–29^, and BH^30,31^. Existing work suggests that SB decreases heart rate^32–37^. But, only a small decrease in heart rate was observed in response to SB in this study. Yet in sum, the experimental data used in this study has provided a quite challenging test for investigating the association between limb BCG-based PTT and BP under a wide range of changes in BP and cardiovascular mechanisms.

### Association between scale PTT and wrist PTT versus BP

Remarkably, PTT calculated with consistent morphological features in the scale and wrist BCG exhibited good association with DP and SP (Table 5 and Figs 3 and 4). The PTT showed the desired inversely proportional response to changes in BP: it decreased when BP increased while it increased when BP decreased (Fig. 3). On the average, when green PPG was used as distal signal, correlation between scale PTT (PTT_S1_ and PTT_S2_) versus DP and SP was ≥0.70 and 0.67, and as well, correlation between wrist PTT (PTT_W1_ and PTT_W2_) versus DP and SP was ≥0.75 and ≥0.76. In addition to good correlation, both scale and wrist PTT achieved promising post-calibration BP error levels (Table 6 and Table 7). Specifically, the accuracy may not appear excellent (especially SP) when each BP measurement is viewed individually. However, noting that an important merit associated with cuff-less BP monitoring is its compatibility for frequent measurement, the random errors in the individual BP measurements may be averaged out^38^. From this standpoint, both scale and wrist PTT achieved post-calibration RMSE levels to attain hypertension screening accuracy comparable to auscultation via measurement averaging (DP ≤8 mmHg and SP ≤12 mmHg)^38^. In addition, both achieved ≤7 mmHg MAE criterion required by the recent IEEE Standard for Wearable, Cuffless Blood Pressure Measuring Devices (IEEE Std 1708™-2014) for DP but not for SP. Comparing all the PTTs examined, PTT_W2_ exhibited the best association with both DP and SP in terms of all the metrics considered in this study.

The arterial path considered in this study mainly involves mid- and small-size vessels (i.e., the subclavian artery, brachial artery, and radial artery, in which the influence of smooth muscle contraction (SMC) is non-trivial). Hence, the imperfect PTT-BP association may be due to SMC rather than alterations in the BP dependence of arterial stiffness. In particular, CP and MA maneuvers may have elicited a large degree of SMC.

It is also emphasized that the PTTs investigated in this study were not calculated randomly via trial and error. In fact, the notable association between the proposed scale and wrist PTT versus BP may be physiologically justified. In regards to the scale PTT, it has been elucidated that the timing associated with the onset of the I wave of the whole-body BCG may correspond to the onset of ascending aortic BP wave^16^. Noting that the scale BCG acquired in this study may be regarded as a whole-body BCG, PTT_S1_ may serve as a viable PTT due to the proximity between the peak of the H_S_ wave and the onset of the I_S_ wave, while PTT_S2_ may as well serve as a viable PTT due to the proximity between the onset and peak of the I_S_ wave, in addition to the robustness in the detection of the peak of the I_S_ wave compared with its onset (in fact, the association between PTT_S2_ acquired with a high-bandwidth force plate and BP has been demonstrated in our prior work^5,21^). In regards to the wrist PTT, the wrist BCG in this study was acquired with an accelerometer. Thus, with a simplifying approximation that the human body is rigid (meaning that the whole-body and wrist movement responses to the heartbeat is exactly the same), the wrist BCG may be viewed as the second derivative of the whole-body BCG. Considering that the whole-body BCG is approximately inverted (i.e., vertically flipped) if differentiated twice, the I_W_ and J_W_ waves may approximately correspond to the H_S_ and I_S_ waves. For this reason, PTT_W1_ and PTT_W2_ may be viewed as equivalent to PTT_S1_ and PTT_S2_ and thus serve as viable PTT.

It is also worth mentioning that the wrist PTT outperformed the scale PTT in all the metrics considered in this study (Tables 5–7 and Fig. 4). Although additional work must be conducted for complete understanding of this observation, this may be attributed to several reasons. First, it may be due to the possible distortion of the whole-body BCG when acquired by the scale due to factors such as (i) dynamic characteristics of the scale and quality of the sensors (i.e., strain gauges) embedded in the scale as well as (ii) phase delay associated with the transfer of the whole-body BCG to the scale caused by the compliance of the human body (indeed, our recent work clearly showed that the scale BCG exhibited a non-negligible subject-dependent phase delay relative to the force plate BCG^17^). In contrast to the scale BCG, the wrist BCG may have been subject to less measurement artifact due to the wide passband of the accelerometer used in this study (400 Hz). Second, it may also be due to the motion artifacts caused by breathing. We speculate that breathing may have more salient influence on the whole-body BCG than the wrist BCG because it originates from the main trunk. Hence, it may be of interest to further investigate the influence of breathing on the BCG signal quality.

### Comparison of wrist PTT and PAT

Both the wrist PTT PTT_W1_ and PTT_W2_ outperformed PAT in all the metrics for both DP and SP (Tables 5–7 and Fig. 4). On the average, when green PPG was used as distal signal, correlation associated with PTT_W2_ was higher than PAT by 15% and 12% for DP and SP, respectively, while RMSE and MAE were smaller than PAT by 24% and 16% for DP, respectively, and 16% and 12% for SP, respectively. For DP, all the metrics for PTT_W2_ were significantly superior to those for PAT. On the other hand, only correlation was significantly superior as far as SP was concerned. Considering that PAT correlates quite well to SP^4^, the efficacy of PTT_W2_ (in the sense that it significantly outperformed all the metrics for DP, plus a certain metric for SP) may still be viewed as promising if not superb.

In addition to its overall efficacy described above, PTT_W2_ exhibited more robust and consistent association with BP across diverse BP-perturbing interventions relative to PAT (Fig. 5). First, the correlation between PAT and BP varied 0.33 for DP and 0.28 for SP across the BP-perturbing interventions employed in this study (CP, MA, SB, and BH), whereas the correlation between PTT_W2_ and BP varied only 0.15 for DP and 0.27 for SP across the same interventions. Second, RMSE and MAE associated with PTT_W2_ were consistently lower than (or at least equal to) the same metrics associated with PAT in all the BP-perturbing interventions for DP and SP (except for SP in MA). On the other hand, PAT showed higher correlation than PTT in MA. It is speculated that the efficacy of PAT in MA may be attributed to the response of PEP to a large increase in heart rate invoked by MA: an increase in cardiac output followed by a large increase in heart rate yielded a (albeit modest) decrease in PEP (Fig. 3), which, when synergistically combined with a decrease in PTT in response to MA, makes PAT more sensitive to change in BP than PTT_W2_. But all in all, PTT_W2_ was more robust and consistent than PAT in terms of association with BP.

In addition to its remarkable performance relative to PAT, wrist PTT may also boast superior convenience to PAT: the measurement of ECG may require conventional electrodes or two-handed user maneuvers (e.g.,^39^), whereas wrist BCG may be passively measured without requiring any user actions. Hence, wrist BCG may turn out to be an attractive alternative to PAT.

### Effect of choice of PPG on PTT-BP association

All in all, green PPG resulted in superior association with BP (both DP and SP) than IR PPG (Tables 5–7). This finding may be attributed to the anatomy of the wrist vasculature and the wavelengths of the green and IR PPG. First, the arterial bed in the back of the wrist is primarily composed of arterioles and capillaries while there is no major large artery passing through the back of the wrist. Second, green PPG is good at capturing capillary blood flow and perfusion at the level of skin, whereas IR PPG is more suited for capturing blood flow and perfusion deep under the skin (where relatively large arteries are often located)^40^. Hence, green PPG may possess superior signal quality than its IR counterpart at the wrist site and may thus be preferred for calculating PTT equipped with close association with BP.

### Study limitations

This study has several limitations. First, the participant pool was not diverse: all were healthy with no explicit indication of cardiovascular disease. Hence, future investigation needs to be conducted to determine if the association between the wrist PTT and BP can be generalized to more diverse subject cohorts, in particular those with cardiovascular disease. Second, PTT was investigated for its association with both DP and SP. In theory, PTT calculated with the diastolic level features may exhibit close association with DP but not necessarily with SP. The wrist PTT proposed in this study, which is built upon the I_W_ and J_W_ waves in the wrist BCG (corresponding to the aortic DP^16^) and the foot of the PPG (corresponding to distal DP), may be a viable surrogate of DP but not necessarily SP. Hence, future investigation needs to be conducted to identify independent and viable wrist BCG-based surrogate of SP. Third, this study was limited to a specific posture (i.e., standing with the arms placed at the side) under minimal movement. The shape of the wrist BCG (and in general any wearable limb BCG potentially) varies with respect to posture, since the change in posture alters the orientation of the sensors embedded in the wearable device^19^. In addition, dynamic limb movement during the postural change may also have influence on the shape of the BCG. Compensation of the impact of postural change and the associated dynamic limb movement on the shape of the wrist BCG may require additional sensors (e.g., inertial sensors) and sophisticated signal processing algorithms to determine the orientation of the wrist. Furthermore, additional consideration is needed if the wrist is artificially supported (e.g., if it is placed on a desk), in which the way the whole-body BCG is transferred to the wrist is altered by the reaction force exerted by the support. Rigorous future work is required to address this challenge to truly enable the wearable limb BCG-based PTT and BP monitoring. Fourth, despite the notable potential of the wrist BCG in ultra-convenient PTT and BP monitoring, convenient PTT-BP calibration still remains an open challenge. Given that artery stiffens with aging and alters PTT-BP relationship, PTT-BP relationship must be continually calibrated. Existing PTT-BP calibration techniques typically involve the measurement of reference BP and PTT during BP-perturbing interventions. However, ideal calibration must not require cumbersome interventions to maximize convenience. Future investigation needs to be conducted for the development of novel convenient PTT-BP calibration techniques. Fifth, this study involved technical weaknesses that may act as obstacles in realizing the BCG-based wearable cuff-less BP monitoring in real world, including (i) the use of the ECG signal for BCG and PPG beat segmentation, (ii) participant-specific adjustment of filtering band, and (iii) visual inspection and manual removal of corrupted BCG and PPG beats. Future work must be conducted to enable ECG-less signal processing as well as to standardize and automate signal processing procedure.

## Conclusions

Close and robust association between the wrist BCG-based PTT and BP was demonstrated. The finding may open up new opportunities for ultra-convenient BP monitoring based on the BCG acquired at limb locations, using, e.g., wristband, armband, and smartphone. Future effort must be invested to (i) the translation of the findings from this study to innovative BP monitoring systems and algorithms applicable to real-world use, as well as (ii) the enhanced physiological understanding of the limb BCG and its relationship to the whole-body BCG, and its application to independent monitoring of DP and SP.
